# Polyhydroxybutyrate Targets Mammalian Mitochondria and Increases Permeability of Plasmalemmal and Mitochondrial Membranes

**DOI:** 10.1371/journal.pone.0075812

**Published:** 2013-09-23

**Authors:** Pia A. Elustondo, Plamena R. Angelova, Michał Kawalec, Michał Michalak, Piotr Kurcok, Andrey Y. Abramov, Evgeny V. Pavlov

**Affiliations:** 1 Department of Physiology and Biophysics, Dalhousie University, Halifax, Novia Scotia, Canada; 2 Centre of Polymer and Carbon Materials, Polish Academy of Sciences, Zabrze, Poland; 3 UCL Institute of Neurology, London, United Kingdom; University of Mississippi, United States of America

## Abstract

Poly(3-hydroxybutyrate) (PHB) is a polyester of 3-hydroxybutyric acid (HB) that is ubiquitously present in all organisms. In higher eukaryotes PHB is found in the length of 10 to 100 HB units and can be present in free form as well as in association with proteins and inorganic polyphosphate. It has been proposed that PHB can mediate ion transport across lipid bilayer membranes. We investigated the ability of PHB to interact with living cells and isolated mitochondria and the effects of these interactions on membrane ion transport. We performed experiments using a fluorescein derivative of PHB (fluo-PHB). We found that fluo-PHB preferentially accumulated inside the mitochondria of HeLa cells. Accumulation of fluo-PHB induced mitochondrial membrane depolarization. This membrane depolarization was significantly delayed by the inhibitor of the mitochondrial permeability transition pore - Cyclosporin A. Further experiments using intact cells as well as isolated mitochondria confirmed that the effects of PHB directly linked to its ability to facilitate ion transport, including calcium, across the membranes. We conclude that PHB demonstrates ionophoretic properties in biological membranes and this effect is most profound in mitochondria due to the selective accumulation of the polymer in this organelle.

## Introduction

Poly-3-hydroxybutyrate (PHB) is a polyhydroxyalkanoate, a polymer that belongs to the polyester class and consists of 3-hydroxybutyrate (HB) units. PHB is ubiquitously present in all living organisms ranging from bacteria to humans. Previous studies have identified two major pools of PHB. The first pool is a long chain or storage PHB, composed of up to 100,000 HB units. This polymer is found predominantly in certain types of bacteria and in these organisms it accumulates in PHB granules under conditions of nutrient limitation, playing a role in carbon and energy storage [1]. The second pool of PHB is a short chain or “complexed” PHB (cPHB), represented by polymers with chain lengths ranging from 2 to 100 monomeric units. The term “complexed” reflects the fact that this type of PHB is normally associated with other biological polymers including proteins and polyphosphates, whereas long chain PHB is normally organized in PHB granules. Unlike long chain storage PHB, cPHB has been found in all living organisms, suggesting it may have an important biological role [2]. Currently the biological functions of cPHB are not well understood and most likely, vary depending on the specific organism and the sub-cellular localization of the polymer. It has been demonstrated that cPHB is likely involved in the regulation of membrane transport. It has been shown that cPHB is directly involved in the formation of bacterial cation selective channels through the formation of a polyphosphate (polyP)/Ca^2+^/PHB complex [3]. Furthermore, recent studies indicate that cPHB is closely associated with the protein part of the bacterial channels KcsA [4] and OmpA [5] and mammalian TRPM8 [6], [7] suggesting a role in controlling the function of these protein channels. Recent studies also suggest that endogenous PHB might play an important role in mitochondrial calcium transport [8]. However, the exact role of PHB in the physiological function of the cell remains poorly understood.

Previous experiments using artificial lipid membranes demonstrated that PHB can induce ion permeability [9], [10]. We hypothesized that this property of PHB can significantly affect ion transporting properties of biological membranes and consequently modify cell function. Herein, we performed experiments in which we studied the effect of synthetic cPHB polymer on the function of live cells and isolated mitochondria. In order to do this we used synthetic fluorophore-labeled cPHB (fluo-PHB). We found that when added to live cells fluo-PHB redistributes into the mitochondria and activates their cation transport. We conclude that in mitochondrial membranes PHB acts as a potent ionophore. Due to the lack of information about the levels of endogenous mitochondrial, PHB effects seen in our study should be considered pharmacological rather than physiological. However, we propose the possibility that endogenous PHB might play a critical role in mitochondrial ion transport.

## Results

### Fluo-PHB Localizes to Mitochondria in Intact Live Cells

**Figure 1:**
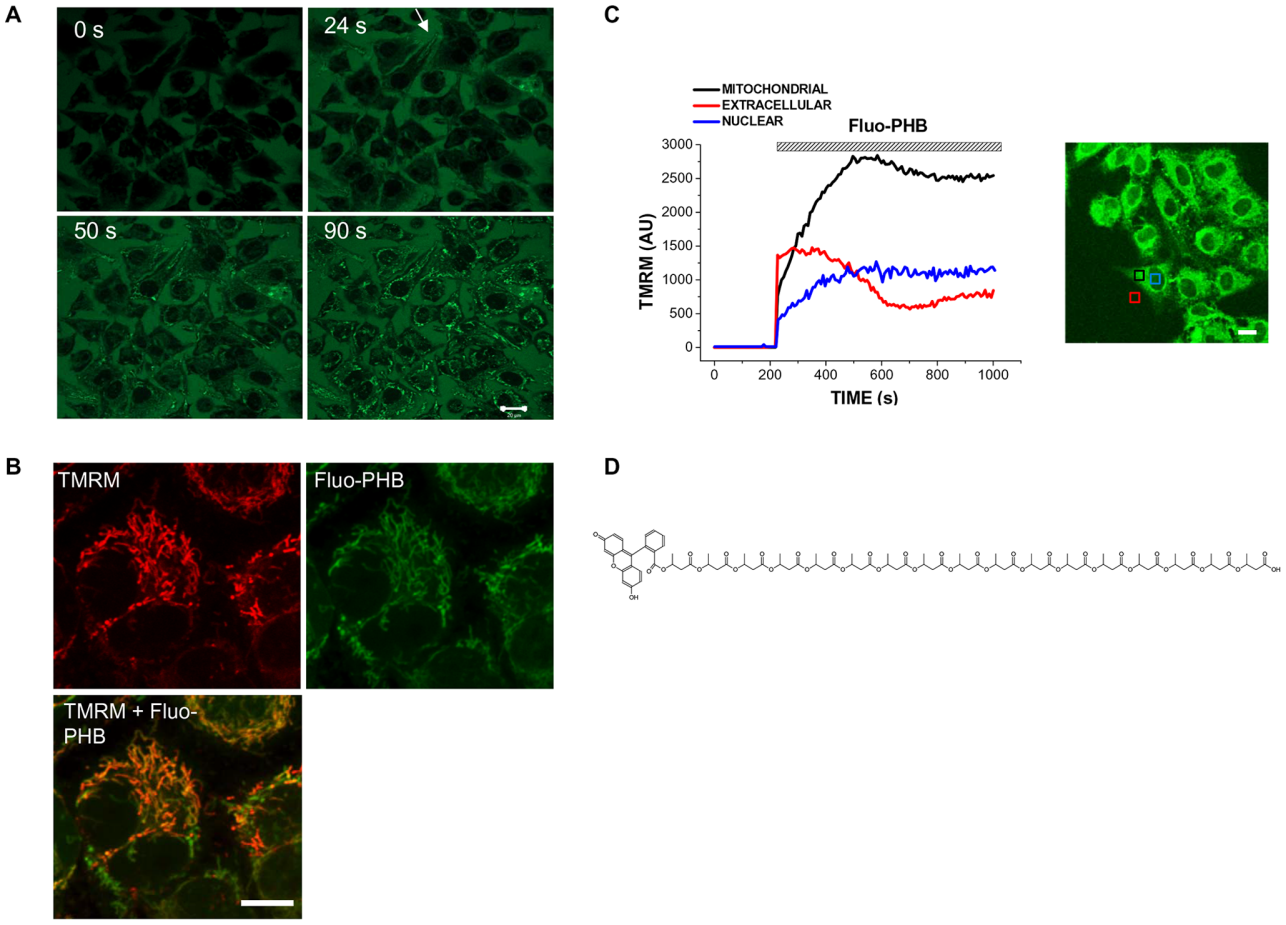
Fluorescein-conjugated PHB (Fluo-PHB) is preferentially distributed in the mitochondria of HeLa cells. A) 1.8 ng/ml fluo-PHB was added to HeLa cells. Times indicated in the panels correspond to acquisition points after fluo-PHB was added. Scale bar 20 µm. B) HeLa were loaded with 25 nM TMRM and with 1.8 ng/ml of fluo-PHB. Scale bar 10 µm. C) Kinetics of distribution of fluo-PHB in the mitochondrial, nuclear and in the extracellular regions. Scale bar 20 µm. D) Chemical structure of fluo-PHB (poly([R]-3-hydroxybutyrate is shown).

**Figure 2:**
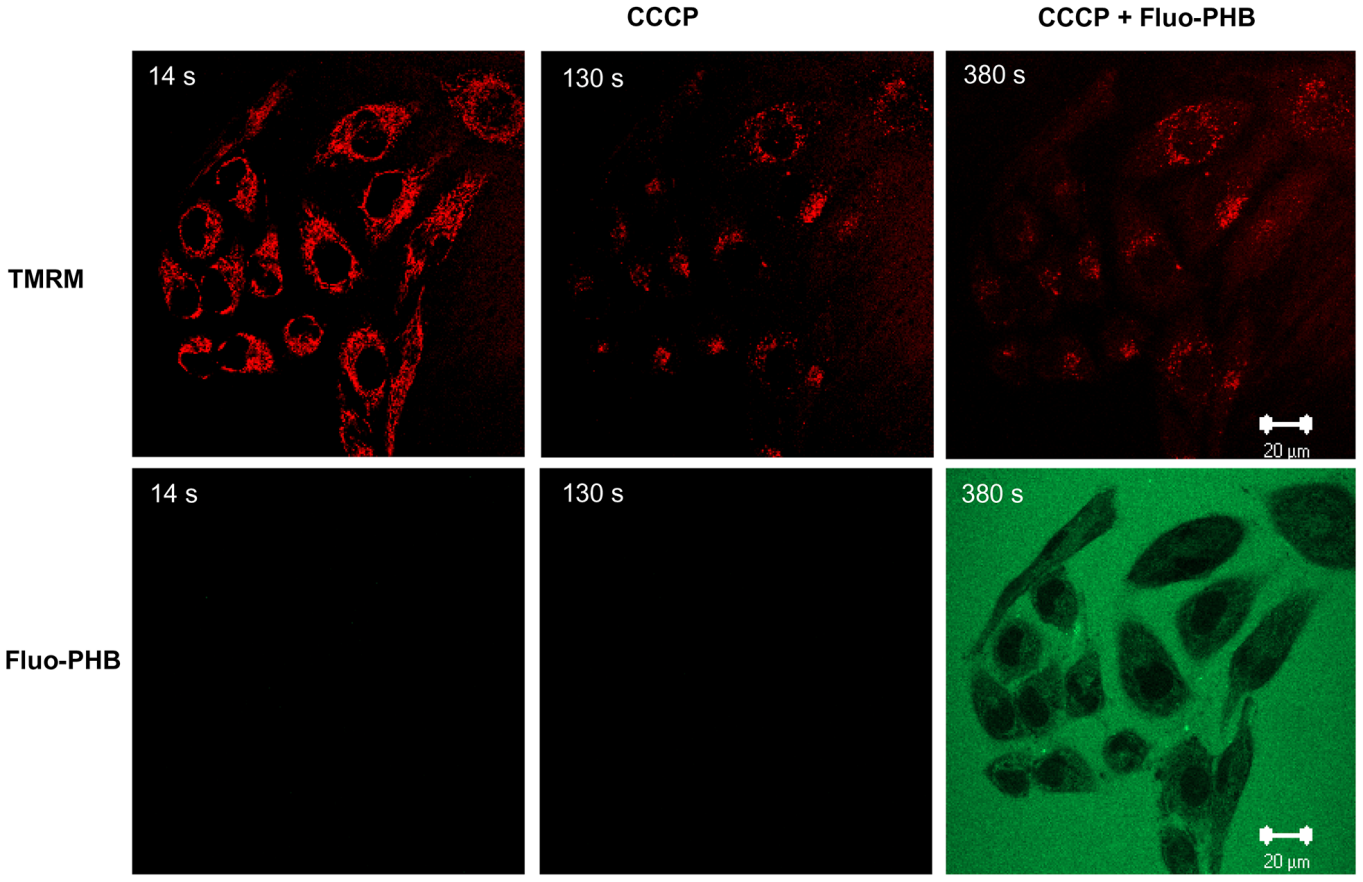
Fluo-PHB is not incorporated into depolarized mitochondria. HeLa cells were loaded with 25 µM CCCP which was followed by the addition of fluo-PHB. This figure shows a representative image before and after fluo-PHB addition. Times correspond to the intervals from the start of the time lapse recording. Scale bar 20 µm.

**Figure 3:**
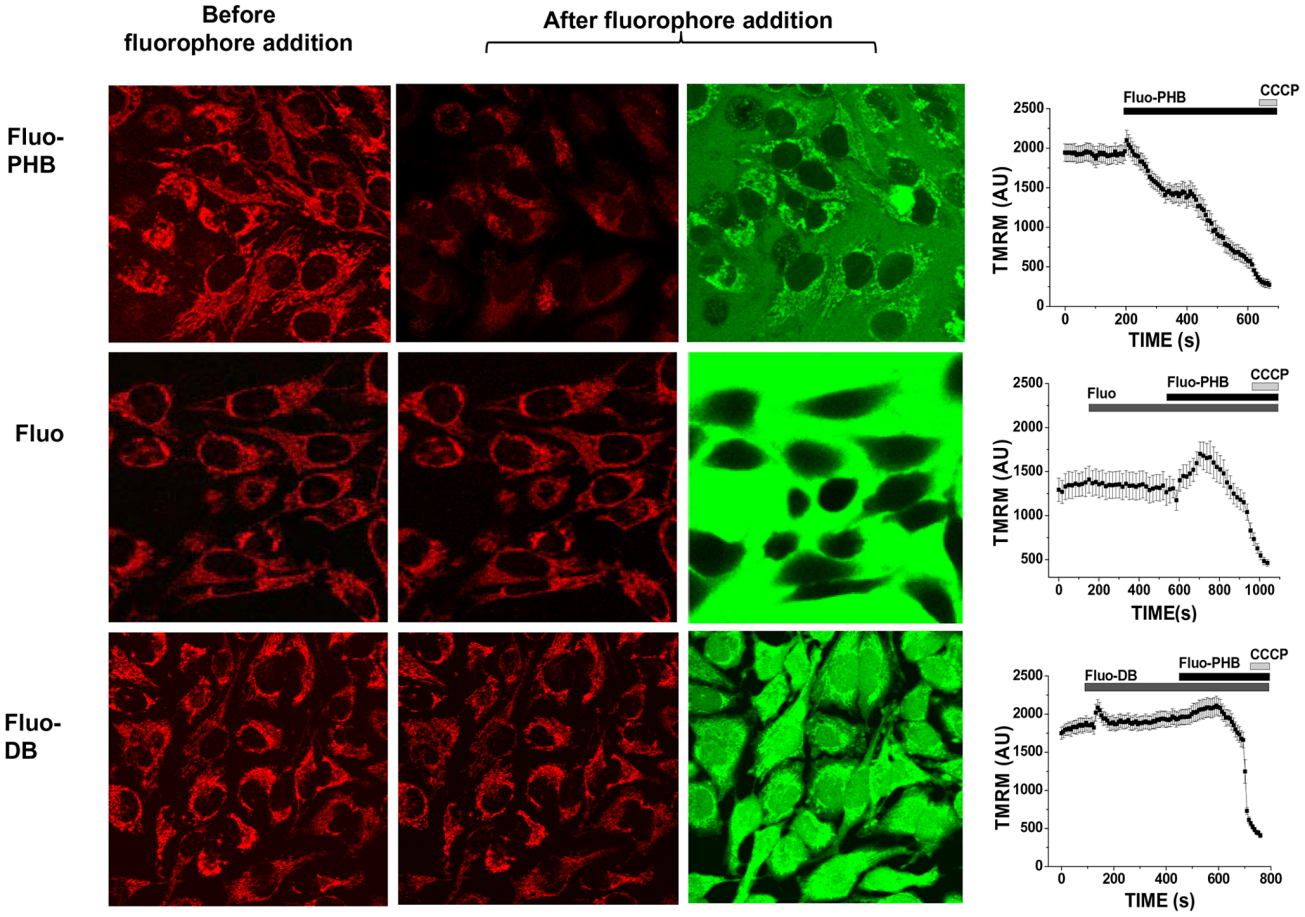
Fluo-PHB but not fluorescein or fluo-DB induces mitochondrial membrane depolarization. HeLa cell loaded with 25/ml of fluorescent probes and imaged with a confocal microscope over time. Left column shows TMRM fluorescence in mitochondria before treatment. Second and third columns show TMRM and fluorescein after addition of the probes. Fluorescein did not distribute inside the cells, while fluo-DB did not show preferential mitochondrial localization. Note that neither fluorescein nor fluo-DB affected mitochondrial membrane potential, which was decreased only in the presence of fluo-PHB. This is represented in the graphs that are in the left column that show TMRM fluorescence in arbitrary units (AU) collected from the mitochondrial regions of the intact cells as a function of time. Scale bar 20 µm.

Taking into account that in eukaryotic cells native PHB is found in mitochondria [11] we hypothesized that mammalian cells possess the specific transporting system which will allow such an accumulation. To test this hypothesis we investigated the distribution of exogenously added PHB in eukaryotic cells using HeLa cells as a model. In our experiments we added fluo-PHB to live cells and monitored its transport and intracellular distribution as a function of time using confocal fluorescent microscopy. The Fluo-PHB preparation contained a mixture of poly([R,S]-3-hydroxybutyrate polymers, their size ranging from 2 to 17 HB units. Previous studies suggest that despite differences in stereochemical configuration this polymer closely resembles the biochemical properties of endogenous poly([R]-3-hydroxybutyrate) [12]. As can be seen from figure 1A and 1C, the application of 1.8 ng/ml of fluo-PHB initially caused an increase in fluorescence in the extracellular media. Within several seconds after the addition, fluo-PHB enters the cell, where it preferentially localized to the mitochondria (Fig. 1A, C). After ninety seconds all the cells in the field (n = 57) showed distinct mitochondrial loading with fluo-PHB (Fig. 1A). We confirmed the mitochondrial localization using the fluorescent indicator for mitochondrial membrane potential TMRM (25 nM). Indeed, as can be seen from Fig. 1B, the TMRM signal overlays with the fluo-PHB signal. At such a low concentration of fluo-PHB mitochondria were able to maintain their membrane potential as indicated by the lack of the change in the TMRM signal (Fig. 1B and Fig. S1). Overall, our data suggest that fluo-PHB can be accumulated by the mitochondria. Importantly, we did not observe fluo-PHB accumulation in the mitochondria when they were depolarized by the addition of an uncoupler such as CCCP (Fig. 2). The active nature of this accumulation appears to require functionally polarized mitochondria, rather than passive diffusion and binding to one or more putative mitochondria targets. It is also noteworthy that, at physiological pH, most fluo-PHB is a negatively charged polymer. Fluo-PHB can carry one or two negative charges due to the presence of phenols which possess acidic properties. This implies that its import into the mitochondria occurs through a specific transporting mechanism. Indeed, due to the high negative membrane potential of functional mitochondria, simple diffusion in intact mitochondria would favor an accumulation of positively rather than negatively charged compounds. An alternative explanation is that fluo-PHB accumulates in mitochondria because of the pH gradient across the mitochondrial inner membrane. In this scenario, diffusion of protonated, uncharged fluo-PHB from the intermembrane space (pH approximately 7.4) to the matrix will result in accumulation of fluo-PHB as the neutral form which deprotonates and is accumulated in the more alkaline matrix (pH approximately 8) due to its lower membrane permeability. In support of this possibility, it is important to point out that fluo-DB molecule, although is found in mitochondria does not demonstrate such a preferential localization (Fig. 3, lower row). This agrees well with the fact that, unlike fluo-PHB, fluo-DB does not contain free carboxylic acid groups which can be protonated or deprotonated. On the other hand, simulation data suggest that even at pH = 7.4 fluo-PHB exists almost exclusively in ionized form. Specifically, our simulation data reveal that pKa1 = pKa-COOH = 4.16 and pKa2 = pKa-OH = 6.42 (for phenolic group). This leads to contributions: [fluo-PHB2-] = 90.50%, [fluo-PHB-] = 9.49% and unionized [fluo-PHB] = 0.01% at pH = 7.4, while [fluo-PHB2-] = 97.43%, [fluo-PHB-] = 2.57% and [fluo-PHB] = 0.00% at pH = 8.0. This argues against the possibility that preferential mitochondrial distribution of fluo-PHB is determined by the protonation of the molecule.

### Fluo-PHB Effects on the Mitochondrial Membrane Potential in Intact Cells

**Figure 4:**
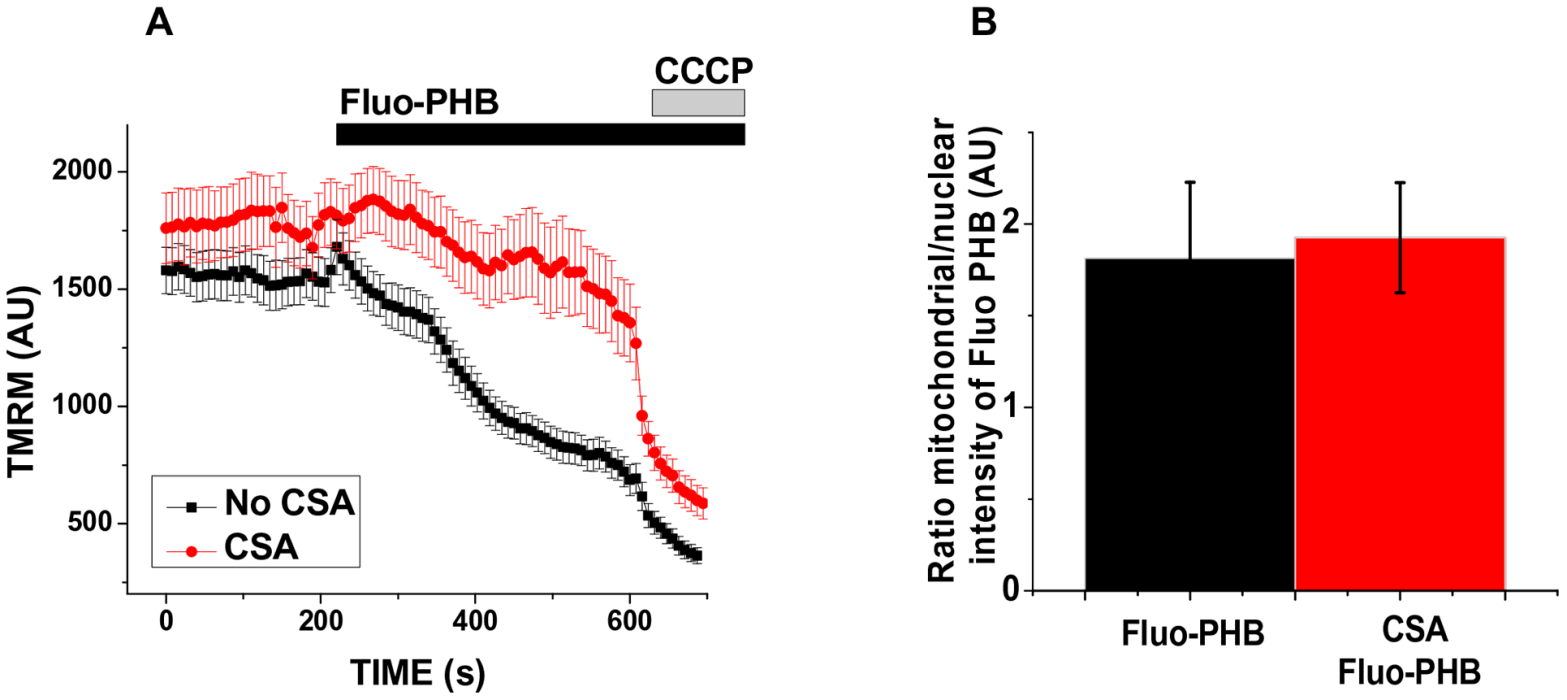
CSA delays fluo-PHB induced mitochondrial membrane depolarization. A) HeLa cells were loaded with 25 nM TMRM and treated (red trace, n = 12) or not (black trace, n = 20) with CSA (1 µM); this was followed by the addition of fluo-PHB (18 ng/ml). Cells were imaged with a laser confocal microscope. Traces show TMRM intensity collected from the mitochondrial regions. B) fluorescence intensity ratios in the presence of fluo-PHB between nuclear and mitochondrial regions of HeLa cells treated with CSA and non-treated control cells. (n = 40 for each group of cells);

**Figure 5:**
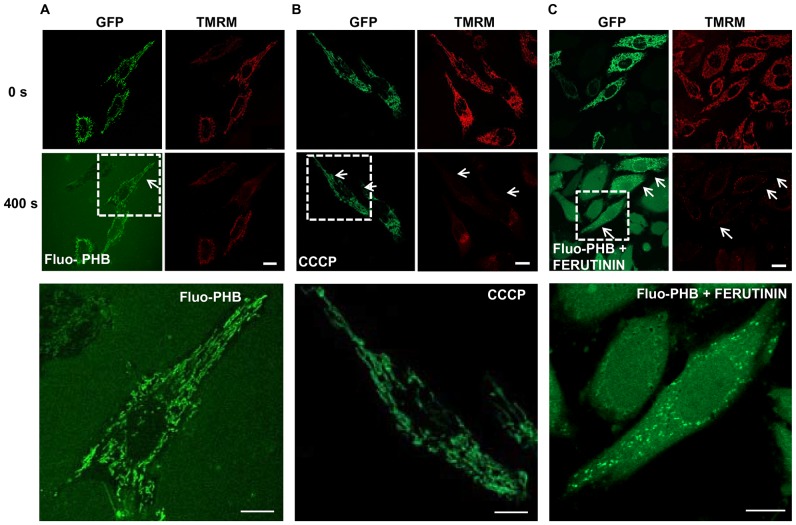
Investigation of the mitochondrial morphology following the addition of fluo-PHB and CCCP. A) fluo-PHB induced mitochondrial membrane depolarization occurs prior to mitochondrial swelling. HeLa cells were transiently transfected with GFP and then loaded with TMRM and fluo-PHB. Arrows point at the region which lost membrane potential but the shape of mitochondria was not changed compared to polarized mitochondria. Images were collected immediately after loading and at 400 s after addition of the Fluo-PHB; B) experimental conditions are similar to those shown in panel A) except in the lower panel the cells were imaged immediately following the addition of 10 µM CCCP. C) HeLa transiently transfected with pMito-GFP were treated with fluo-PHB (18 ng/ml) and after 400 s these cells were treated with ferutinin (25 µM) showing typical swelling of the mitochondria. Scale bar is 20 µm.

Next, we investigated the functional consequences of PHB accumulation in mitochondria. We found that a 10-fold higher concentration of fluo-PHB (18 ng/ml) added to HeLa cells caused a profound decrease in the TMRM fluorescence by 84±10% (n = 13; p<0.001), indicating significant depolarization of the mitochondrial membrane (Fig. 3, upper row, see control experiment showing TMRM fluorescence change in response to mitochondrial depolarization by CCCP on figure S4). We should note that synthetic unmodified PHB did not have any effect on TMRM fluorescence, when added from the stock dissolved in DMSO. As previously proposed this is likely due to the fact that free PHB is poorly soluble in water and has limited bioavailability [9]. To confirm that the observed effects are caused by PHB polymer rather than by fluorescein we performed control experiments using free fluorescein (Fig. 3, middle row) and monomeric dibutyrate labeled with fluorescein (fluo-DB) (Fig. 3, lower row). When treated with free fluorescein, the green fluorescent signal was localized in the extracellular media. This indicates that fluorescein cannot permeate cell membrane on its own. Addition of fluorescein did not affect mitochondrial membrane potential. On the other hand, fluo-DB was able to cross the cell membrane but did not preferentially accumulate in the mitochondria. The observed abilities of the tested compounds to permeate cell membranes should be considered in the context of their calculated distribution coefficients (LogD), which are estimated to be the following: 5.99 for fluo-DB (in this particular case logD = logP since it cannot be ionized); −1.19 (pH = 7.4) and −1.78 (pH = 8.0) for quinonoid and 3.86 (pH = 7.4) and 3.80 (pH = 8.0) for lactone form of fluorescein and −1,62 (pH = 7.4) and −1,95 (pH = 8.0) for fluo-PHB. This indicates that the ability of the molecule to freely permeate the membrane was not a sufficient explanation for the preferential mitochondrial localization, and further supports the specificity of the mitochondrial fluo-PHB transport. Compared to control cells, fluo-PHB-induced mitochondrial depolarization was significantly less profound in the cells treated with 1 µM CSA [an inhibitor of the mitochondrial permeability transition pore (PTP)]. There was a 52±3% decrease in the control cells (n = 19) compared to 18±11% in the CSA treated cells (n = 10; p<0.01; Fig. 4A). However, in our experiments, the presence of CSA did not affect the degree of the fluo-PHB accumulation in the mitochondria (n = 40, Fig. 4B), suggesting that the CSA effect was directly involved in inhibiting the ability of fluo-PHB to induce depolarization, as opposed to affecting the distribution of fluo-PHB. Interestingly, although the CSA inhibition suggests that the mitochondrial depolarization occurred through a mechanism related to the activation of PTP, this depolarization occurred prior to any significant change in the length of the mitochondria, as monitored by the mitochondrially targeted GFP protein fluorescence (n = 10, Fig. 5A). This effect was similar to the depolarization induced by the addition of 10 µM CCCP-(a mitochondrial uncoupler) (n = 10, Fig. 5B). These observations were confirmed by the morphometric measurements of the length of the mitochondria shown on Fig. 5A and B. The mitochondria did not show any significant changes in the mitochondrial average length following membrane depolarization by either CCCP (4.7±1.8 µm prior to CCCP addition (n = 55, individual mitochondria) and 3.8±1.2 µm following CCCP addition (n = 50, individual mitochondria from the same cell), or fluo-PHB (5±2 µm prior to fluo-PHB addition (n = 50, individual mitochondria) and 5.1±1.7 µm following fluo-PHB addition (n = 50, individual mitochondria from the same cell). This was in significant contrast to the dramatic change in the mitochondrial shape induced by the addition of the known activator of mPTP – ferutinin. As can be seen in figure 5C and figure S2, the addition of ferutinin caused the change of the mitochondrial shape from elongated, which is typical for normal mitochondria, to spherical, which is typical for swollen organelles with induced high conductance mPTP (see also more detailed image at figure S2). We should also note that in some experiments we observed mitochondrial fragmentation following addition of CCCP but this fragmentation was significantly delayed by the order of minutes and was secondary to membrane depolarization.

### Effect of Fluo-PHB on Calcium Transport

**Figure 6:**
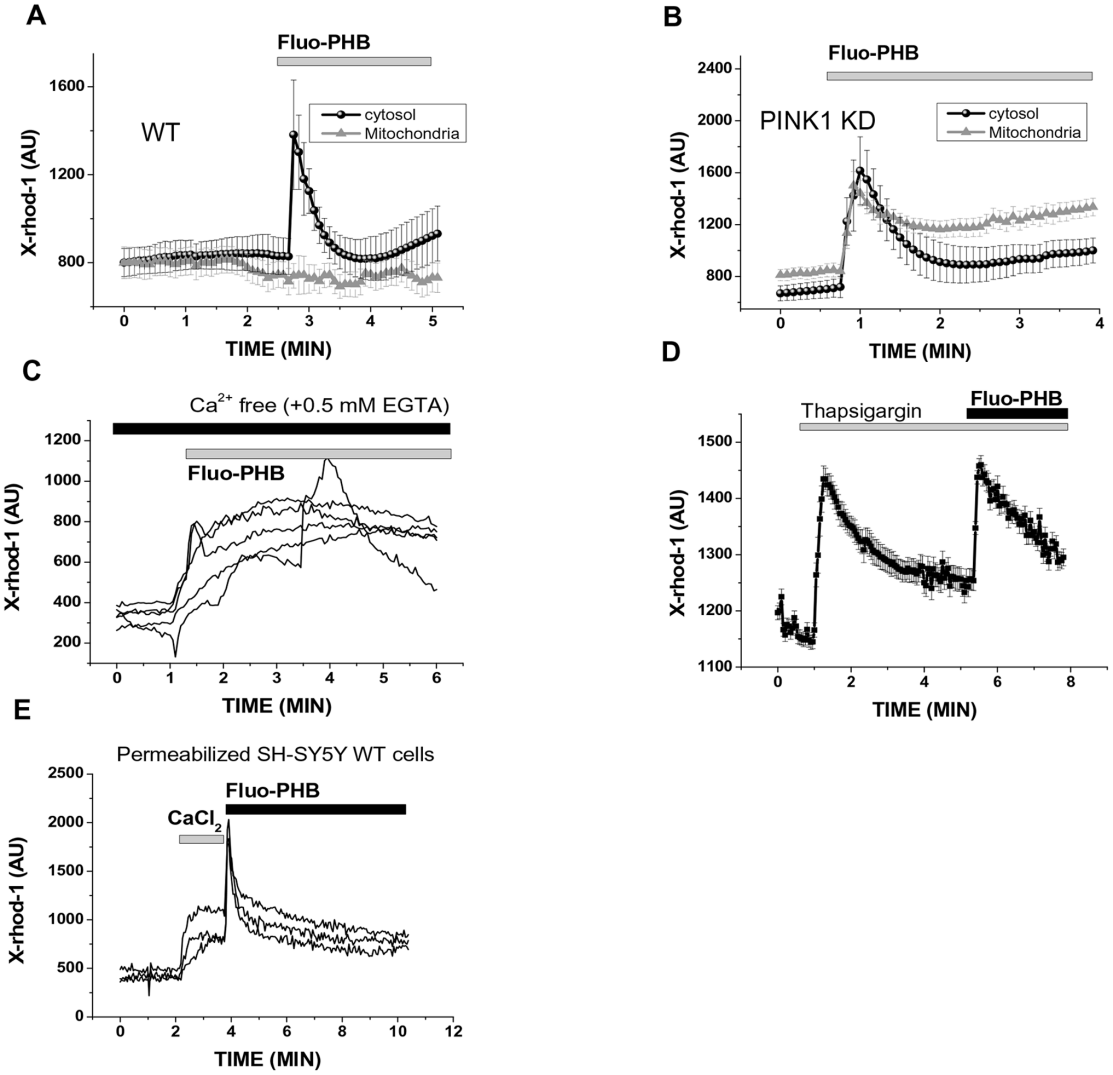
The effect of fluo-PHB on [Ca^2+^]c and [Ca^2+^]m. Application of fluo-PHB (1.8 ng/ml) to wild type (WT) A) PINK1 knockdown B) human dopaminergic neuroblastoma cell line (SH-SY5Y) induced increase in [Ca^2+^]c, but [Ca^2+^]m changed only in cells with PINK1 deficiency. The measurements of the fluorescence shown in panels A and B were obtained from analyzing fluorescence from cytoplasmic and mitochondrial regions of the cells (see also Fig. S6 for details). Fluo-PHB-induced calcium signal in SH-SY5Y WT cells is not prevented by cell incubation in Ca^2+^-free medium (+0.5 mM EGTA) (C); 0.5 µM thapsigargin (D)– each trace represents fluorescence measured from individual cytoplasmic region of the individual cell; E) Fluo-PHB induced Ca^2+^-rise in mitochondria of permeabilized cells. Each trace on the panel E represents measurements of the fluorescence from individual mitochondrial region of interest.

Previous studies showed that PHB is capable of transporting calcium ions across lipid bilayers [2]. To test the ability of PHB to modulate mitochondrial and cytosolic calcium transport, we used calcium sensitive fluorescent probe X-rhod-1 which can be monitored by observing changes in fluorescence intensity in the mitochondrial and cytoplasmic regions. For these experiments we used WT and the PINK1 knock-out SH-SY5Y cells. The use of a cell line with genetically altered mitochondrial calcium transport allowed us to perform more detailed investigation of the calcium signal changes induced by fluo-PHB. In control experiments we found that, similarly to other cell types tested, fluo-PHB redistributes primarily to the mitochondria (data not shown). We found that the addition of fluo-PHB to wild type (WT) SH-SY5Y cells causes a transient increase in cytosolic calcium (by 506±45 AU; n = 18) but we did not detect any significant changes in the mitochondrial calcium concentration (Fig. 6A, see also cells figure S6 as example of X-rhod-1 staining). However, the addition of fluo-PHB to the PINK1 knock-out SH-SY5Y cells caused significant increases not only of cytoplasmic but also of mitochondrial calcium concentration (by 550±60 AU in cytosol; n = 21; by 330±35 AU in mitochondria; Fig. 6B). Taking into account that PINK1 knock-out cells have an impaired mitochondrial calcium efflux due to the reduced activity of the Ca^2+^/Na^+^ exchanger [13], [14], we propose that fluo-PHB can cause a moderate stimulation of calcium transport which can be compensated by the exchanger activity. Overall, these experiments demonstrate that fluo-PHB can directly stimulate calcium transport across biological membranes. However, this stimulation is very moderate considering that it can be compensated by the activity of mitochondrial Ca^2+^/Na^+^ exchanger.

In order to investigate the mechanism of PHB-induced calcium signal, we applied fluo-PHB to WT SH-SY5Y cells in a Ca^2+^ free medium (plus 0.5 mM EGTA). The absence of calcium in the recording medium did not prevent the PHB-induced [Ca^2+^]_c_ changes, but changed the shape of the signal (n = 38 cells; Fig. 6C). The incubation of the WT SH-SY5Y cells with 0.5 µM thapsigargin (an inhibitor of ER Ca^2+^ pump) depleted Ca^2+^ from the ER but did not prevent the PHB induced [Ca^2+^]_c_ changes (n = 51, Fig. 6D). In earlier experiments we have established that this concentration of thapsigargin applied to SH-SY5Y cells completely depletes ER of Ca^2+^ in 5 minutes period as tested by the lack to ATP – stimulated Ca^2+^ signal following thapsigargin application [14]. This strongly suggests that fluo-PHB has no preferences for the source of Ca^2+^. These results can be explained by a channel or ionophoretic activity of this compound, but cannot exclude an effect on plasma membrane ion channels. To avoid the possible effect of the plasma membrane channels we permeabilized the cells in pseudo-intracellular solution containing 20 µM digitonin [14]. The application of 5 µM CaCl_2_ induced an increase in [Ca^2+^]_m_ (n = 59 cells; Fig. 6E). The application of fluo-PHB induced a further increase of calcium in the mitochondria, which strongly suggests that PHB works as an electrogenic Ca^2+^-ionophore or activates mitochondrial Ca^2+^-uniporter. Considering the effects of PHB on plasma, ER and mitochondrial membranes we suggest that this compound has ionophoretic properties in biological membranes. This compound transports Ca^2+^ into mitochondria through an electrogenic mechanism, as oppose to Ca^2+^-ionophores ionomycin and A23187 which use Ca^2+^ gradients, and can transport calcium out of mitochondria when gradient is higher in this organelle [15].

### Effect of Fluo-PHB on the Mitochondrial Ion Transport

**Figure 7:**
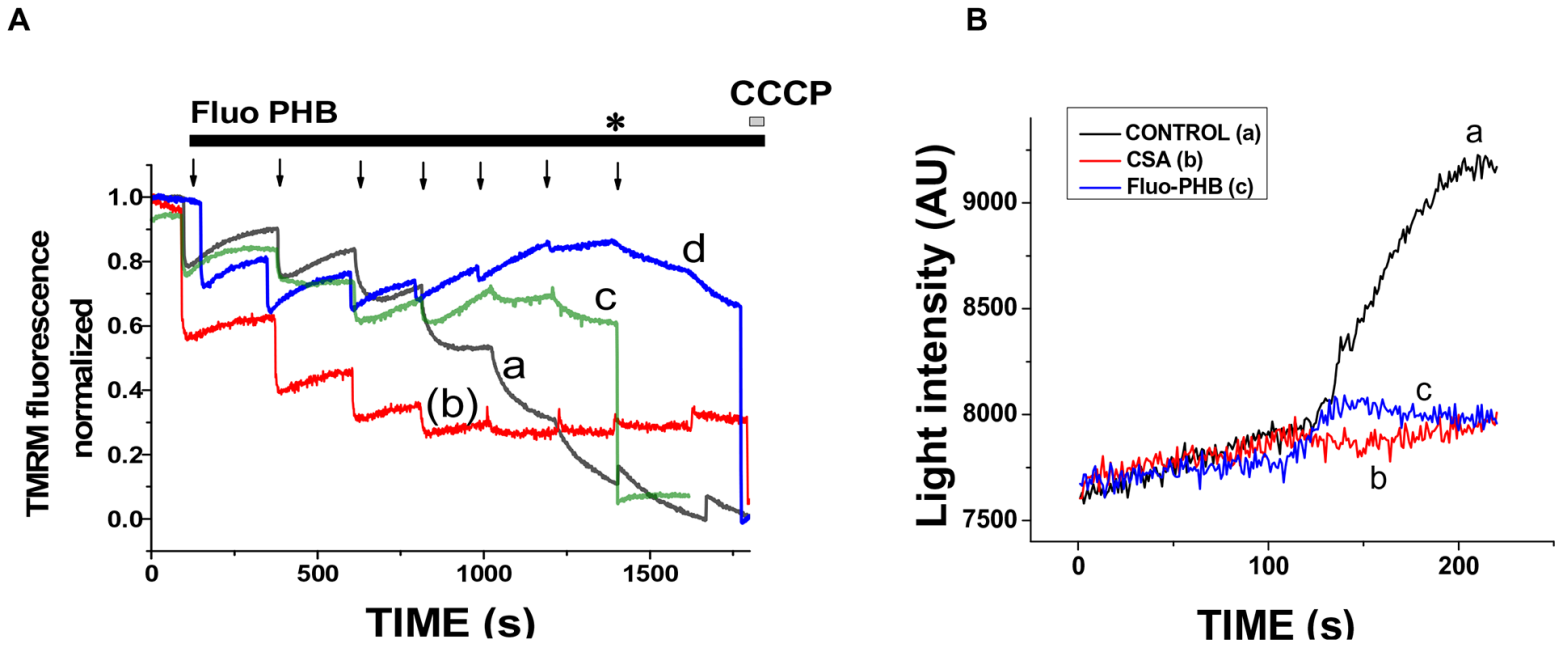
Effect of fluo-PHB on the membrane potential of isolated mouse liver mitochondria. A) Isolated mouse liver mitochondria were suspended to the concentration of 1 mg/ml in 2 ml of solution containing 150 mM KCl and 10 mM NaCl pH 7.4 in the presence of rotenone (1 µM) and succinate (5 mM) and loaded with 0.2 µM TMRM. Following 30 min of equilibration period the intensity of the fluorescence was measured with a spectrofluorimeter with excitation set at 546 nm and emission at 590 nm. During the experiment increased amounts of fluo-PHB were added to the cuvette. Each arrow corresponds to the addition of 1.8 ng/ml of fluo-PHB. Each trace corresponds to a different treatment either with fluo-PHB alone (trace a) or in the presence of EGTA (1 mM) (trace b); RuR (1 µM) (trace c) or CSA (1 µM) (trace d) and then addition of CCCP. * CCCP was added to RuR (trace c) experiment at this point. Fluorescence was normalized to be equivalent to 1 at the beginning of the experiment and 0 in the presence of CCCP under conditions of complete membrane depolarization. B) isolated mitochondria in ICM (150 mM KCl, 10 mM NaCl pH 7.4 in the presence of 1 µM rotenone and 5 mM succinate) show no swelling when treated with the same amounts of fluo-PHB that induces depolarization (18 ng/ml). Isolated mitochondria were treated with 50 µM calcium to induce swelling (control), this swelling was inhibited when the isolated mitochondria were pre-incubated with 1 µM of CSA.

To further investigate the ability of PHB to increase membrane permeability and to elucidate the details of this phenomenon we performed fluorometric assays of the mitochondrial membrane potential on isolated mouse liver mitochondria using a TMRM probe. This assay relies on the fact that when the TMRM is added to the suspension of isolated mitochondria, it redistributes differently between the media and the mitochondrial matrix, depending on whether the mitochondria have membrane potential or are depolarized [16]. This results in differences in fluorescent spectra of mitochondria with different values of membrane potential. Figure S3A shows an overlay of fluorescence spectra of fully energized mitochondria and mitochondria depolarized by CCCP. This experimental setting allows the detection of changes in the mitochondrial membrane potential by measuring fluorescence at wavelength of 546 nm. Figure 7A presents results of that assay. To make a direct comparison possible, the raw fluorescent data (see figure S3, B) were normalized with the fluorescence reading of fully energized mitochondria assigned value of “1” and the fluorescence reading of completely depolarized mitochondria, in the presence of CCCP assigned value “0”. Normalized traces on Figure 7A show measurements of the mitochondrial membrane potential using 0.2 µM TMRM in a salt based solution containing 150 mM KCl and 10 mM NaCl pH 7.4 in the presence of rotenone (1 µM) and succinate (5 mM). We found that in this solution the addition of increasing concentrations of fluo-PHB initially induced partial mitochondrial membrane depolarization eventually leading to the complete membrane depolarization (Fig. 7A, trace (a)). The initial depolarization was not sensitive to the addition of CSA – the inhibitor of PTP (Fig. 7A, trace (d)), EGTA (Fig. 7A, trace (b) – a chelator of calcium, and ruthenium red – an inhibitor of the mitochondrial calcium uptake (Fig. 7A, trace (c)). This suggests that the partial depolarization is not related to the activity of the PTP. Due to the fact that PHB can transport ions other than calcium including monovalent ions we hypothesize that the depolarization effect that was not blocked by CSA was due to the stimulation of either sodium or potassium transport. This indicates that partial depolarization caused by fluo-PHB is likely caused by the PHB-induced stimulation of the mitochondrial ion transport. Interestingly, at least in the case of CSA and ruthenium red we were able to detect a significant change in TMRM fluorescence following addition of CCCP. This suggests the possibility that complete membrane depolarization might be related to the activation of PTP. The interpretation of the fluorescence changes in the presence of EGTA was somewhat complicated due to the small relative response to the addition of CCCP. Next, we tested if fluo-PHB can induce mitochondrial swelling, which is indicative of the activation of high-conductance mPTP. We performed experiments in which we used light transmittance assay in isolated mitochondria as a measure of their swelling. In these experiments addition of Ca^2+^ to isolated energized mitochondria causes their large amplitude swelling, which can be inhibited by CSA (inhibitor of mPTP). As can be seen from figure 7B the addition of fluo-PHB in the concentration of 18 ng/ml, which is expected to cause mitochondrial depolarization did not induce significant mitochondrial swelling. We interpret these data as further evidence that despite CSA sensitivity fluo-PHB does not induce high-conductance mPTP.

**Figure 8:**
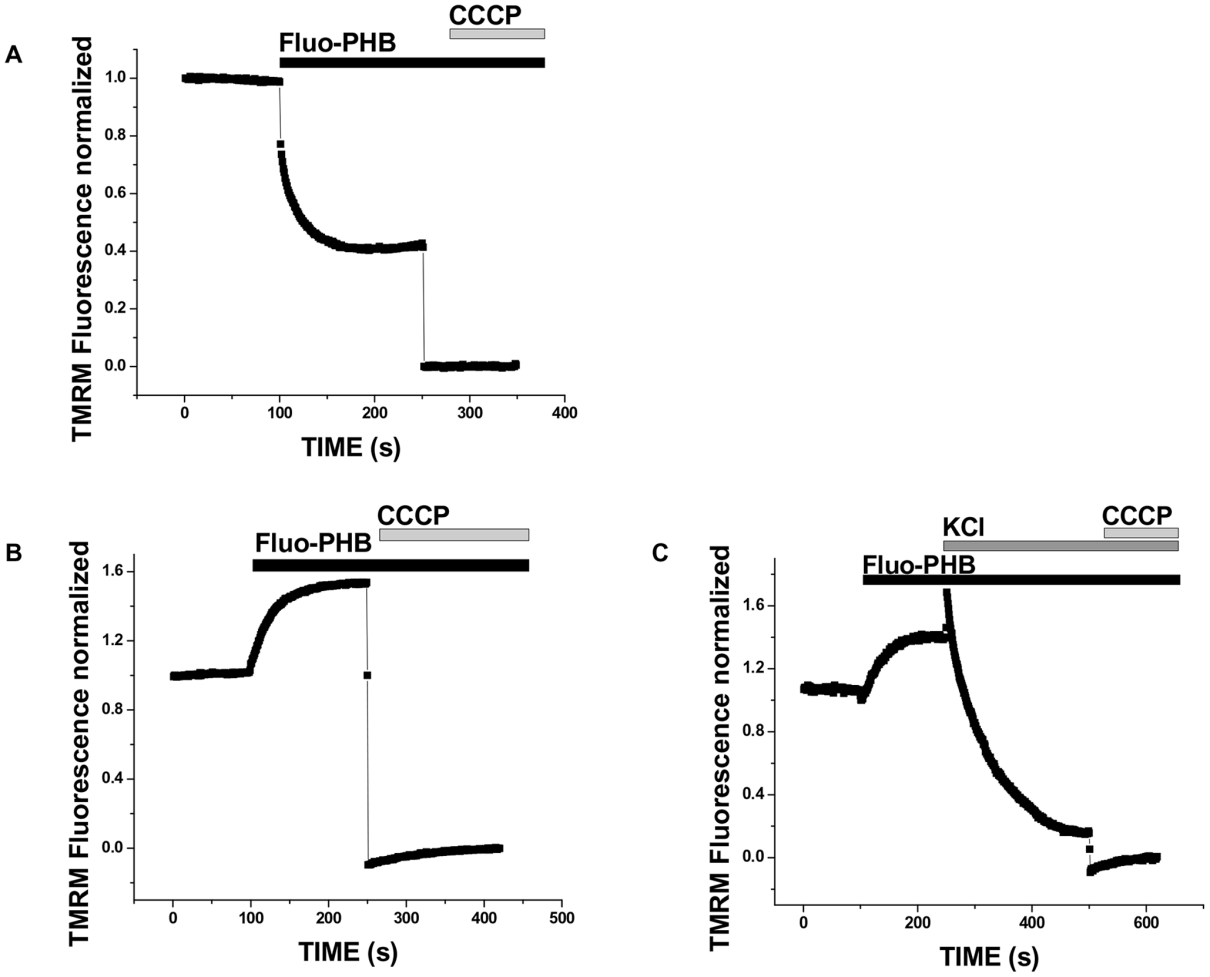
Effect of fluo-PHB on mitochondrial membrane potential in a recording solution with sodium and potassium ions substituted by NMDG. Isolated mitochondria were loaded with 0.2 µM TMRM and fluo-PHB (18 ng/ml) was added either in ICM (a) or NMDG without sodium and potassium ions (b) or followed by addition of KCl (50 mM) (c).

To further test the interpretation that fluo-PHB induces depolarization due to the stimulation of ion transport we studied the effect of fluo-PHB in isolated mitochondria when potassium and sodium ions were replaced with NMDG. NMDG is a positively charged ion which is significantly larger and is not transported across the membranes as easily as sodium and potassium. As can be seen from figure 8B (see also figure S5) under conditions of NMDG ion substitution no depolarization was seen (compare to figure 8A collected using standard ICM solution). Interestingly, we even observed some decrease of fluorescence following fluo-PHB addition (see figure S5), however we cannot say conclusively whether this increase reflects increase in the membrane potential or is an artifact of the system. Importantly, addition of KCl in the presence of fluo-PHB caused membrane depolarization (Fig. 8C) confirming the interpretation that effects of depolarization are linked to the activation of ion transport. Finally, we should note that in control experiments the addition of KCl in the absence of fluo-PHB induced only moderate depolarization presumably due to the activation of the endogenous potassium transporting systems (data not shown).

## Discussion

Fluo-PHB enters intact cells and is predominantly accumulated in the mitochondria. This fluo-PHB accumulation leads to the induction of mitochondrial membrane depolarization. Overall, our data are consistent with the idea that the main effect linked to the action of fluo-PHB is due to increased permeability of the membrane to ions. This increase in permeability can occur by two possible mechanisms: 1) a selective membrane permeabilization, which allows for the transport of only certain positive ions, notably calcium and potassium and/or 2) a non-selective mechanism, likely related to the activation of the mitochondrial calcium induced PTP. The exact properties of fluo-PHB interactions with the membrane as well as specific ion transporting characteristics likely depend on its interactions with specific membrane proteins. It should be noted that fluo-PHB is a negatively charged polymer. However, it can accumulate in the energized mitochondria despite the presence of a high negative (approximately −180 mV) membrane potential. This suggests that mitochondria contain an active membrane transport system which can allow accumulation of fluo-PHB. Another possibility for preferential mitochondrial accumulation might be related to differential protonation states of fluo-PHB due differences in pH in cytoplasm and mitochondrial matrix. However, we consider this unlikely due to the fact that at pH values found in the cytoplasm and mitochondria over 99% of fluo-PHB is expected to be deprotonated. Fluo-PHB interaction with specific membrane components appears to play an important role based on the fact that it is unlikely to occur by passive diffusion across lipid bilayer. While a regulatory role of membrane proteins and perhaps other components might be very important, we propose that the ability of fluo-PHB to cause an increase in membrane permeability is directly linked to its ionophoretic function. Indeed, earlier studies with a similar polymer demonstrated that it can act as an ionophore and provide transport of cations across artificial lipid bilayers [2], [17]. Although the exact molecular mechanisms of such transport are currently unknown, we note that a polyester structure is able to provide the conformation which will permit such transport. Indeed, PHB is a polymer composed of many alternating hydrophobic and hydrophilic groups [2], [18]. This polymer can form complexes with metal ions and fold with the hydrophobic groups facing the hydrophobic environment found in the lipid bilayer. A similar configuration is typical for other biological ionophores, for example valinomycin and ferutinin. These ionophores are typically found in bacterial organisms and plants, respectively [19], [20]. Interestingly, the existence of natural ionophores in mitochondrial membranes was demonstrated several decades ago [21] but PHB has never been considered as a candidate. On the other hand, previous studies document detection of PHB in various types of eukaryotic organisms, including mammalian mitochondria [11], [22]. Importantly, the roles of PHB in mitochondria have never been addressed before. The only exception is the functional study, which demonstrated that mitochondrial PHB of rat liver might form a part of the calcium induced permeability transition pore (PTP) [23]. Our finding that PHB-induced mitochondrial membrane depolarization is delayed in the presence of the PTP inhibitor CSA is consistent with the idea that PHB might make an important contribution to the activation of PTP. Furthermore, it is possible that in addition to PTP-related pathology, PHB can play important physiological roles. One of the possibilities involves a role of PHB in slow ion uptake. It is known from experiments on isolated mitochondria that these organelles have the ability to allow passage of various ions, most notably Ca^2+^, K^+^ and Na^+^ [24]. Many channel candidates have been proposed to fulfill this task. However, it has been shown that transport of the same ion can occur at dramatically variable rates (see for example [25]). We hypothesize that PHB can provide Ca^2+^ uptake by a mechanism similar to ionophore which involves relatively slow events of ion binding [9] rather than fast free diffusion through the ion selective pore as in channel transport [26] and, by doing so, may be responsible for the slow ion uptake.

It should be noted that in the current investigation we were not able to address directly the role of the endogenous mitochondrial PHB. One of the key obstacles is that the current experimental approaches do not allow to measure and track PHB in living cells. Thus, we focused on the studies of PHB synthetic derivative - fluo-PHB. The key limitation in using fluo-PHB includes the uncertainty about the effects of added PHB on the levels endogenous polymer. Thus at the moment we cannot say conclusively to which extend the results of our study are physiologically relevant. Although the question about the origin of endogenous mitochondrial PHB remains open and requires further investigation, several possibilities can be considered. Firstly, studies of bacterial organisms indicate that PHB metabolism is very closely linked to the energy metabolism and activity of the TCA cycle [1]. It is possible that PHB is directly synthesized inside the mitochondria. Alternatively, recent studies suggest that lipophilic granules found in mammalian cells contain PHB [27], which potentially can be recruited into the mitochondria. Finally, significant amounts of PHB have been found in the extracellular media which can be highly variable in healthy individuals and increased in diabetes [28]. Considering that, according to our data, PHB can be efficiently transported into mitochondria, we propose that extracellular PHB might exert significant effects on mitochondrial function.

In conclusion, we have found that PHB can stimulate cation transport across mitochondrial membrane. We hypothesize that this stimulation is directly related to the ionophoretic abilities of PHB. Taking into account that PHB is found in mitochondria of mammalian organisms we propose that PHB might serve the role of endogenous ionophore and play an important physiological and/or pathological role in mitochondrial ion transport.

## Materials and Methods

### Cell Line

HeLa and the SH-SY5Y wild-type and PINK1 knockout [14] cells were maintained in high glucose Dulbecco’s modified Eagle’s medium (DMEM, Gibco) supplemented with 10% FBS (Invitrogen) and 1% Penicillin/Streptomycin (Invitrogen). Cells were cultured at 37°C in a humidified tissue culture incubator containing 5% CO_2_ in the air.

We purchased pMito-GFP, which encodes a fusion protein consisting of a mitochondrial targeting sequence derived from the precursor of subunit VIII of human cytochrome c oxidase and the GFP from Aequorea coerulescens (Clontech, Mountain View, CA).

### Fluo-PHB Synthesis

Fluo-PHB (3-O-[Oligo-(3-hydroxybutyrate ester)] fluorescein was synthesized via anionic polymerization of β-butyrolactone. Briefly, polymerization initiated with fluorescein sodium salt was carried out in a solution of DMSO ([BL]o 1 mol dm^−3^) at 23°C. Progress of the reaction was measured by FTIR spectroscopy. When the reaction was completed the product was dissolved in water and extracted with dichloromethane. Pure product was isolated and purified as described previously [29]. The final product was characterized with GPC and ESI-MS techniques. For the experiments, stocks were diluted in DMSO and used at different concentrations from a 1.8 ng/ml final concentration in the dish to 18 ng/ml.

### Partition Coefficient (LogD) Calculations

LogD values were calculated using ChemAxon Marvin Calculator Plugin (Marvin 6.0.3, 2013, http://www.chemaxon.com).

### Reagents

Tetramethylrhodamine methylester (TMRM) (Invitrogen). Fluorescein (Fluka, Cat # 32615); fluorescein dibutyrate (Cat # RES4017F-A101X) were purchased from Research Organics, Inc, Cleveland, OH. Uranine (fluorescein sodium salt) (Cat # 28803 Fluka) was used as purchased. β-butyrolactone (98%) (Cat # 219126 Sigma Aldrich) was purified and dried just before using as described previously [30].

### Confocal Microscopy

HeLa cells were incubated with 25 nM TMRM for 30 minutes in a HEPES-buffered salt solution (HBSS) composed of (mM): 156 NaCl, 3 KCl, 2 MgSO_4_, 1.25 KH_2_PO_4_, 2 CaCl_2_, 10 glucose and 10 HEPES; pH adjusted to 7.35 with NaOH and then the different reagents were added at different concentrations. Fluo-PHB was used either at 1.8 ng/ml or 18 ng/ml. Fluorescein and fluorescein conjugated-dibutyrate were loaded at similar concentrations. Images were obtained using a 510 CLSM (Zeiss,Thornwood, NY) equipped with a META detection system and a 40X oil-immersion objective. The 488 nm Argon laser line was used to excite fluorescein, which was measured using a bandpass filter from 505–550 nm. Illumination intensity was kept to a minimum (at 0.1–0.2% of laser output) to avoid phototoxicity and the pinhole was set to give an optical slice of ∼1 µm. TMRM was excited using the 543 nm laser line and fluorescence measured using a 560 nm longpass filter. For measurements of Δψ_m_, cells were loaded with 25 nM tetramethylrhodamine methylester (TMRM) for 30 minutes at room temperature and the dye was present during the experiment. TMRM is used in the redistribution mode to assess Δψm, and therefore a reduction in TMRM fluorescence represents Δψ_m_ depolarisation. Effect of the addition of CCCP in a concentration, which induces complete mitochondrial membrane depolarization on TMRM fluorescence is shown on figure S2. All data presented were obtained from at least 5 coverslips and 2–3 different cell preparations.

For measurements of [Ca^2+^]c and [Ca^2+^]m, cells were loaded for 30 min at room temperature with 5 µM X-Rhod-1 and 0.005% pluronic acid in a HEPES-buffered salt solution (HBSS) composed of (mM): 156 NaCl, 3 KCl, 2 MgSO_4_, 1.25 KH_2_PO_4_, 2 CaCl_2_, 10 glucose and 10 HEPES; pH adjusted to 7.35 with NaOH. X-Rhod-1 probe was chosen due to the lack of fluorescent interference between this probe and fluorescein based fluo-PHB compound. Fluorescence intensity was measured in mitochondrial and cytoplasm Regions of Interest (see also Fig. S6 for details).

For experiments using permeabilized cells, cells were exposed to 20 µM digitonin in a “pseudo-intracellular” solution consisting of (in mM) 135 KCl, 10 NaCl, 20 HEPES, 5 pyruvate, 5 malate, 0.5 KH_2_PO_4_, 1 MgCl_2_, 5 EGTA, and 1.86 CaCl_2_ (to yield a free [Ca^2+^] of ∼100 nM.) using online calculator available at: http://maxchelator.stanford.edu/CaEGTA-TS.htm [31].

Mitochondrial length was measured using ImageJ software (National Institutes of Health, USA). To do this images collected by confocal microscopy at 40X magnification with resolution of the scanning area of 1024×1024 and were analyzed in ImageJ (NIH). The length was approximated as a distance between the terminal ends of the individual mitochondrion. Results of these measurements are reported as an arithmetic average of the lengths of individual organelles and SD.

### Membrane Potential Measurements in Isolated Mitochondria

Mouse liver were homogenized on ice with a dounce homogenizer in mitochondrial isolation buffer (70 mM sucrose, 230 mM Mannitol, 5 mM HEPES-KOH, pH 7.4) and mitochondria were isolated by differential centrifugation [32].

Isolated mitochondria were loaded with 0.2 µM TMRM in either ICM (120 mM KCl, 10 mM NaCl, 1 mM KH_2_PO_4_, 20 mM HEPES, 2 mMgCl_2_, pH 7.4 or in 130 mM N-Methyl glucamine-(NMDG), 1 mM NaH_2_PO_4_, 2 mM MgCl_2_, pH 7.3. Fluo-PHB was added to the cuvettes at final concentrations of 1.8 ng/ml, 3.6 ng/ml, 5.4 ng/ml, 7.2 ng/ml, 9 ng/ml, 10.8 ng/ml, 12.6 ng/ml, 14.4 ng/ml. Membrane potential was monitored by measuring the fluorescence intensity at excitation wavelength 546 nm using an emission detection wavelength of 590 nm with a Quantamaster-4 Spectrofluorimeter (PTI, Birmingham, NJ) and data was analyzed with FelixGX (PTI).

Representative traces shown on Fig. S3 b were collected from the same preparation of isolated mitochondria. These are representative of at least 3 independent experiments performed using mitochondrial isolation from different animals.

### Isolated Mitochondria Swelling

Isolated mitochondria in ICM (120 mM KCl, 10 mM NaCl, 1 mM KH_2_PO_4_, 20 mM HEPES, 2 mMgCl_2_, pH 7.4) buffer were treated either with 50 µM CaCl_2_ alone or in combination with 1 µM CSA and with 18 ng/ml fluo-PHB and the transmittance was recorded at 540 nm with a Quantamaster-4 Spectrofluorimeter (PTI, Birmingham, NJ), and data was analyzed with FelixGX (PTI).

### Statistical Analysis

Data was generated from a minimum of 3 independent experiments, using a minimum of 20 cells/experiment. Statistical analysis was performed with the aid of Origin 8 (Microcal Software Inc., Northampton, MA, USA) software. Means expressed ± the standard error of the mean (s.e.m.).

## Supporting Information

Figure S1 **Co-localization of TMRM and fluo-PHB.** HeLa cells were loaded with 25 nM TMRM and 1.8 ng/ml fluo-PHB. Scale bar, 20 µM.
(TIF)

Figure S2 **HeLa cells from** **figure 5C** **after treatment with 18 ng/ml of fluo-PHB and 25 µM ferutinin.** Scale bar: 20 µM.
(TIF)

Figure S3 **Use of TMRM to measure membrane potential in isolated mitochondria.** A) Isolated mitochondria were loaded with 0.2 µM TMRM and the excitation spectra were collected at 590 nm emission (black trace). Note shift to the left when CCCP is added (red trace). B) Effect of fluo-PHB on isolated mouse liver mitochondria, raw data. Isolated mitochondria were treated with increasing amounts of fluo-PHB alone (trace a) or in the presence of EGTA (trace b), 1 µM CSA (trace c), or ruthenium Red (trace d). At the end of each experiment CCCP was added to achieve complete membrane depolarization. Note that concentration of TMRM used in experiments with isolated mitochondria is higher comparing to the concentration used in the intact cells due to the characteristics of the method. In case of the confocal imaging, low concentration of TMRM allows to directly monitor dye redistribution to and from mitochondria. On the opposite, in the isolated mitochondria the assay detects integral fluorescence from the cuvette, which includes both TMRM fluorescence inside and outside of the mitochondria. In this case fluorescent spectra of the TMRM inside mitochondria is different from the fluorescent spectra of TMRM outside. In these settings higher concentration of TMRM needs to be used [16].
(TIF)

Figure S4 **Effect of the addition of the CCCP to cells on the TMRM fluorescence of the mitochondria.** HeLa cells loaded with 25 nM TMRM were treated with 10 µM CCCP, note the abrupt decrease of fluorescence upon membrane depolarization.
(TIF)

Figure S5 **Effect of fluo-PHB on isolated mouse liver mitochondria.** The same experimental data as shown in figure 8 but presented in absolute units of fluorescence.
(TIF)

Figure S6 **Representative image of SH-SY5Y loading with calcium sensitive probe X-Rhod-1.** The regions with increased red signal correspond to the mitochondria.
(TIF)
