# Supplementary figures and images for: Polyhydroxybutyrate Targets Mammalian Mitochondria and Increases Permeability of Plasmalemmal and Mitochondrial Membranes
Source: PLoS One. 2013 Sep 23;8(9):e75812. doi: 10.1371/journal.pone.0075812 (PMC3781052; doi:10.1371/journal.pone.0075812)

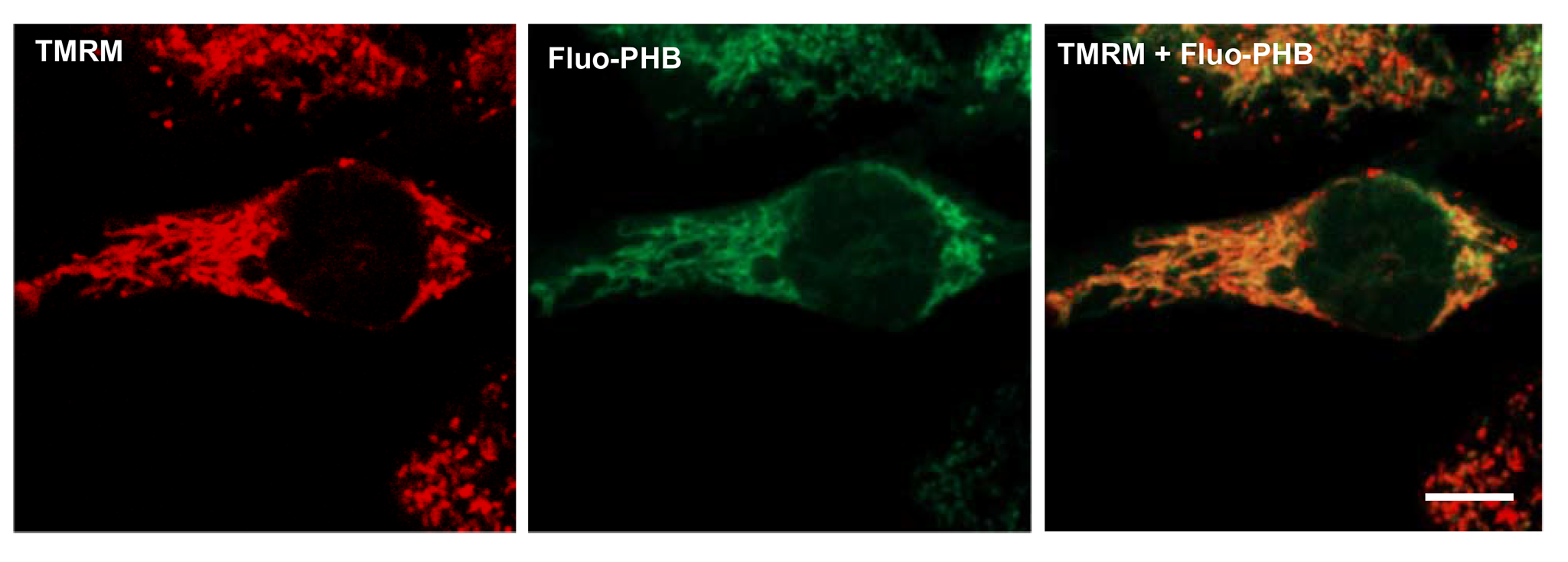

Supplement: Figure S1 — Co-localization of TMRM and fluo-PHB. HeLa cells were loaded with 25 nM TMRM and 1.8 ng/ml fluo-PHB. Scale bar, 20 µM. (TIF) [file pone.0075812.s001.tif]

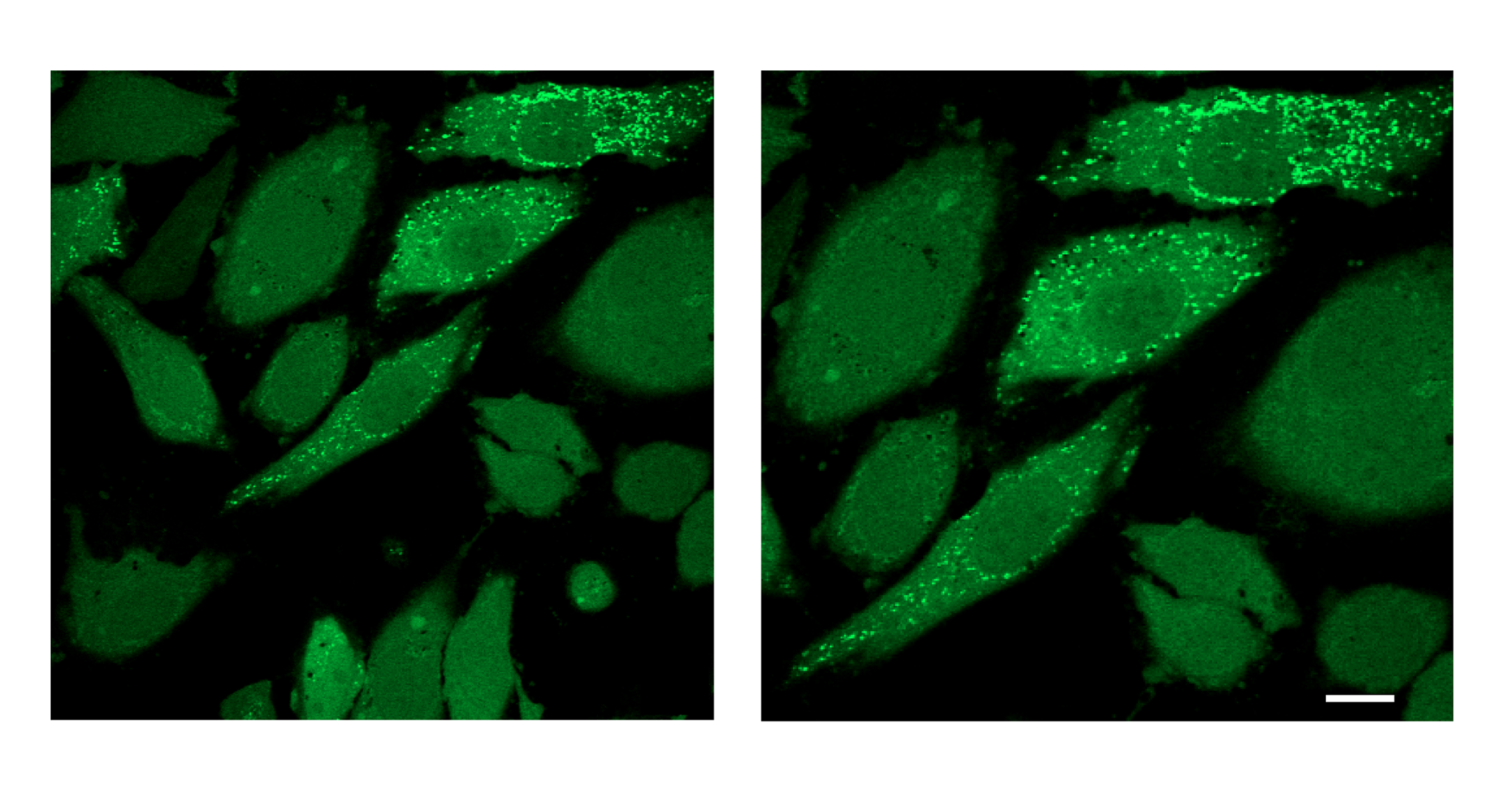

Supplement: Figure S2 — HeLa cells from figure 5C after treatment with 18 ng/ml of fluo-PHB and 25 µM ferutinin. Scale bar: 20 µM. (TIF) [file pone.0075812.s002.tif]

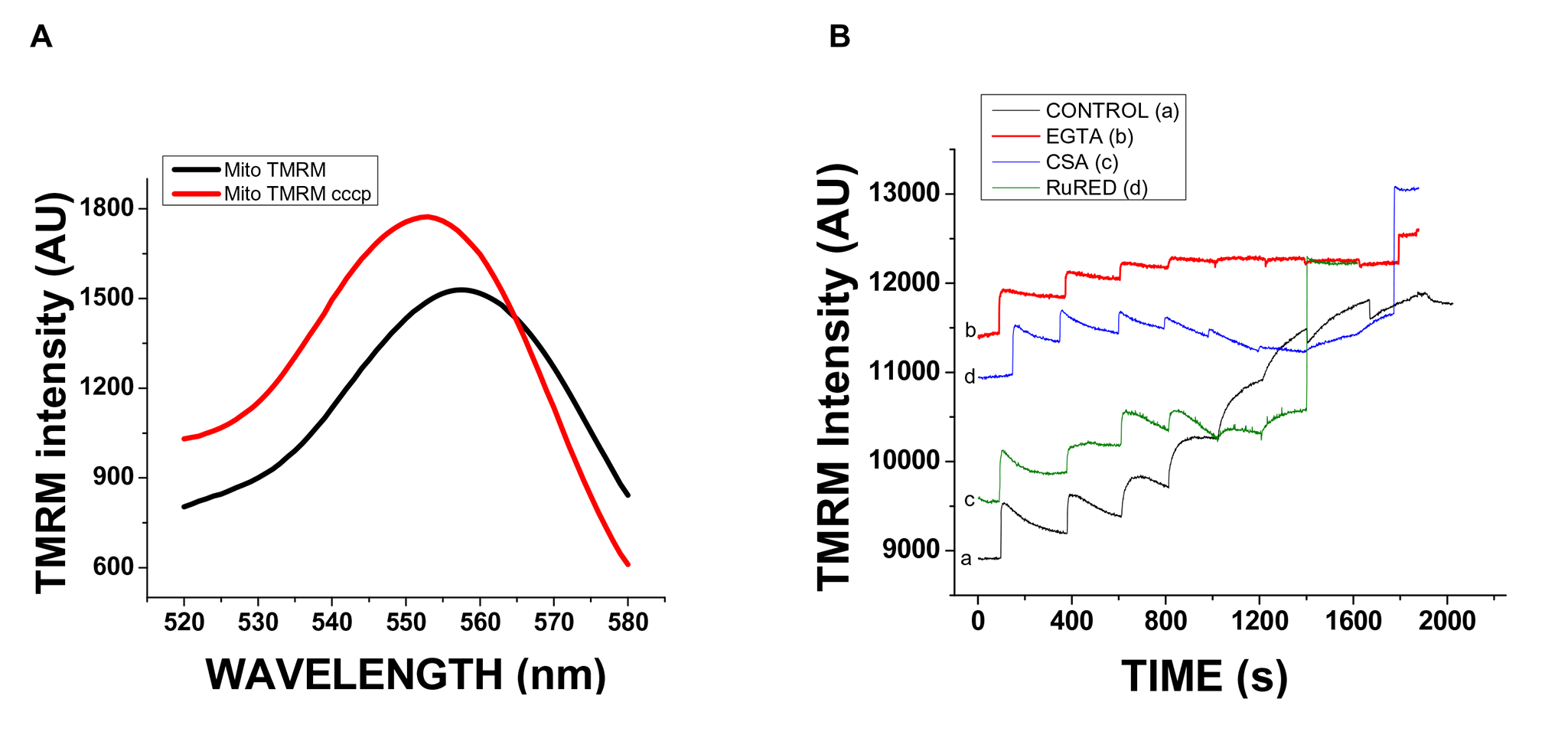

Supplement: Figure S3 — Use of TMRM to measure membrane potential in isolated mitochondria. A) Isolated mitochondria were loaded with 0.2 µM TMRM and the excitation spectra were collected at 590 nm emission (black trace). Note shift to the left when CCCP is added (red trace). B) Effect of fluo-PHB on isolated mouse liver mitochondria, raw data. Isolated mitochondria were treated with increasing amounts of fluo-PHB alone (trace a) or in the presence of EGTA (trace b), 1 µM CSA (trace c), or ruthenium Red (trace d). At the end of each experiment CCCP was added to achieve complete membrane depolarization. Note that concentration of TMRM used in experiments with isolated mitochondria is higher comparing to the concentration used in the intact cells due to the characteristics of the method. In case of the confocal imaging, low concentration of TMRM allows to directly monitor dye redistribution to and from mitochondria. On the opposite, in the isolated mitochondria the assay detects integral fluorescence from the cuvette, which includes both TMRM fluorescence inside and outside of the mitochondria. In this case fluorescent spectra of the TMRM inside mitochondria is different from the fluorescent spectra of TMRM outside. In these settings higher concentration of TMRM needs to be used [16]. (TIF) [file pone.0075812.s003.tif]

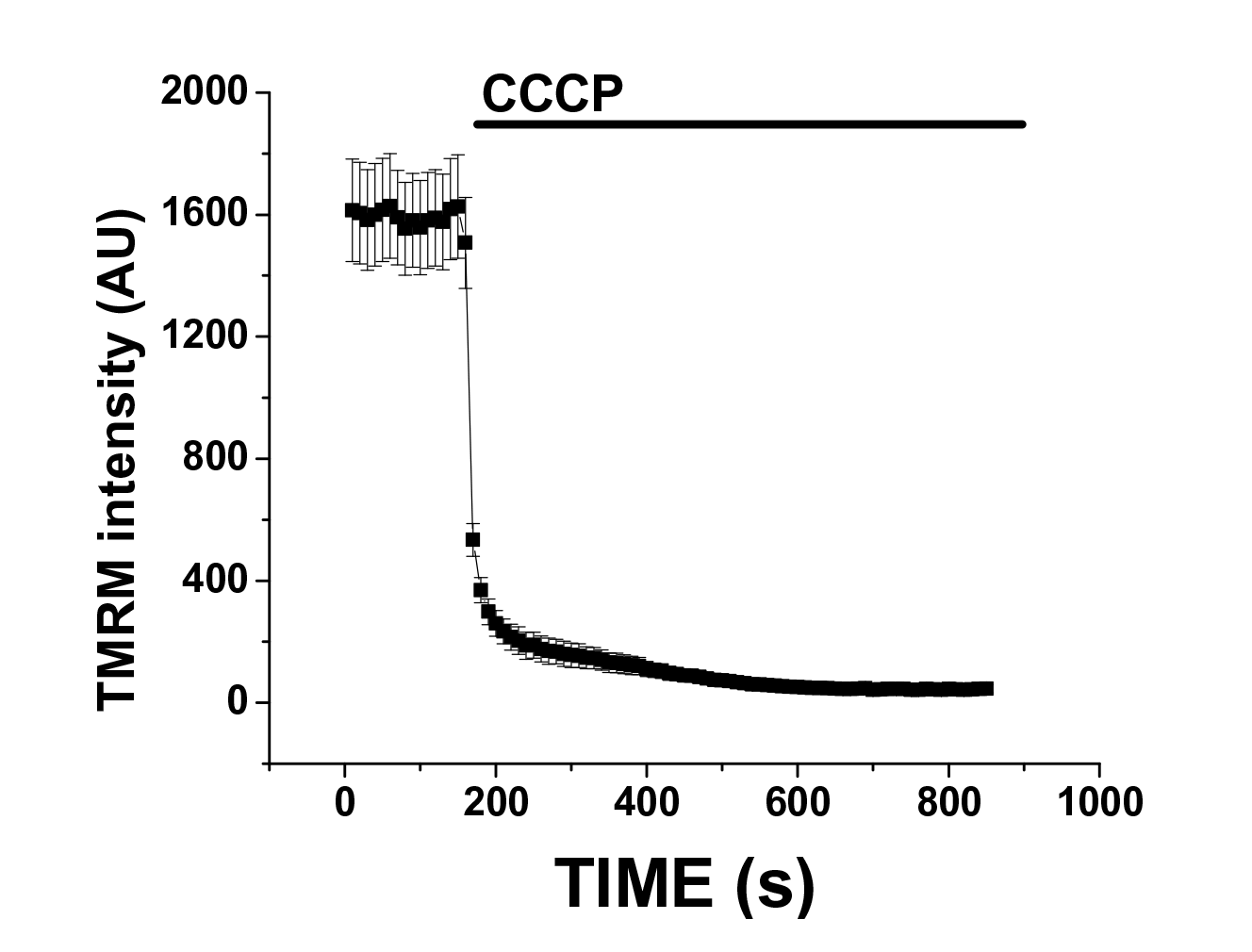

Supplement: Figure S4 — Effect of the addition of the CCCP to cells on the TMRM fluorescence of the mitochondria. HeLa cells loaded with 25 nM TMRM were treated with 10 µM CCCP, note the abrupt decrease of fluorescence upon membrane depolarization. (TIF) [file pone.0075812.s004.tif]

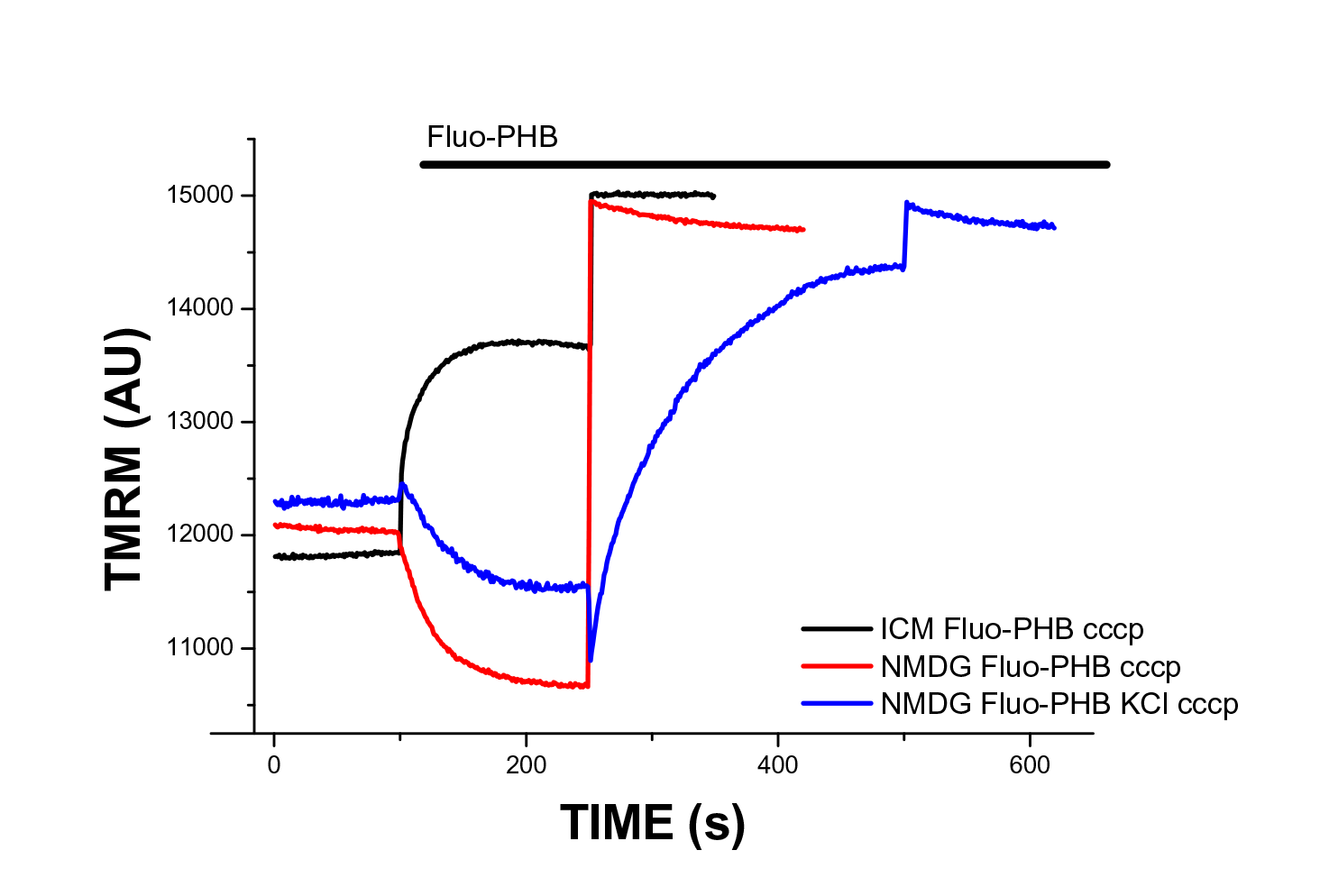

Supplement: Figure S5 — Effect of fluo-PHB on isolated mouse liver mitochondria. The same experimental data as shown in figure 8 but presented in absolute units of fluorescence. (TIF) [file pone.0075812.s005.tif]

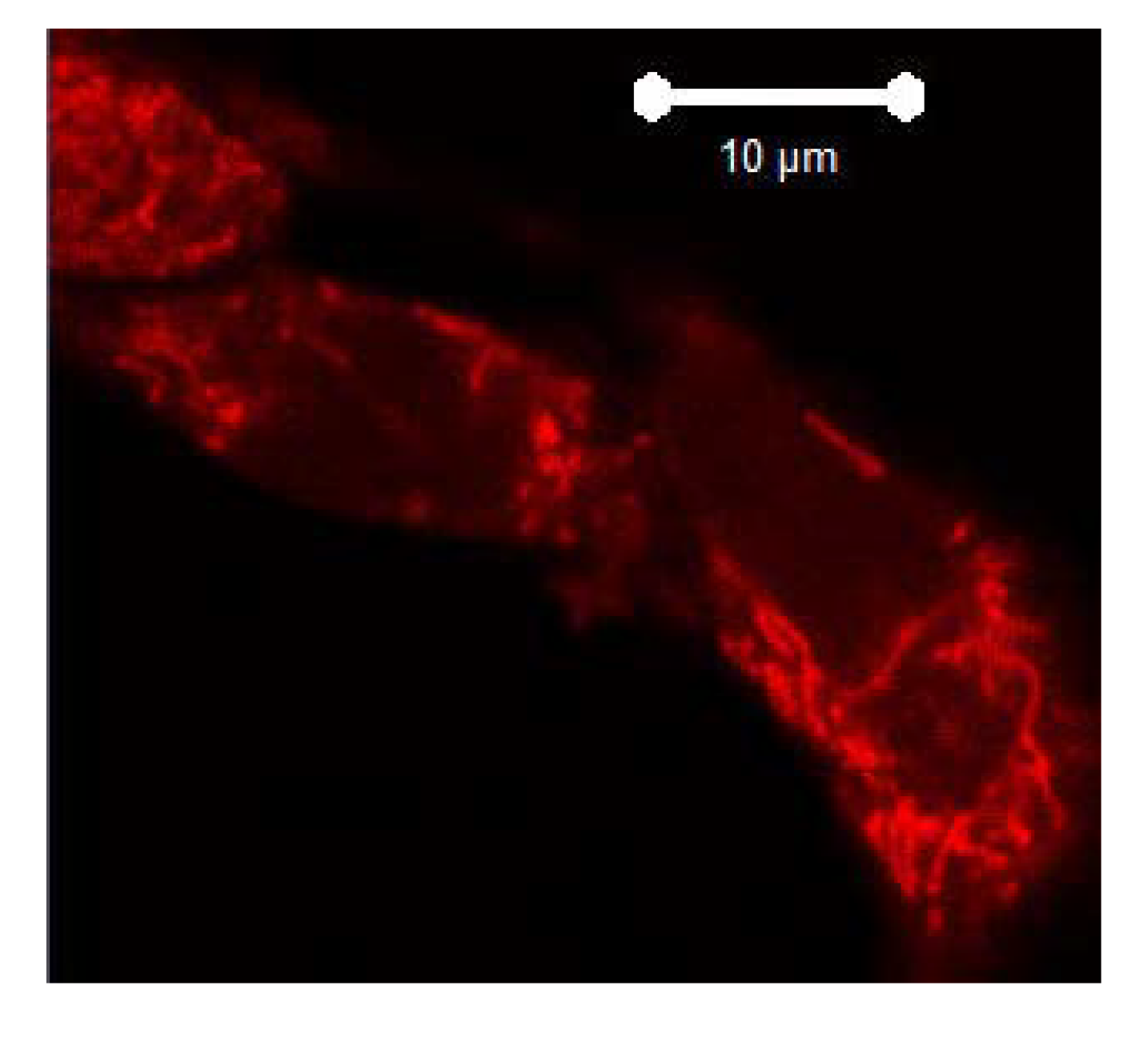

Supplement: Figure S6 — Representative image of SH-SY5Y loading with calcium sensitive probe X-Rhod-1. The regions with increased red signal correspond to the mitochondria. (TIF) [file pone.0075812.s006.tif]
